# Prevalence of Body Dysmorphic Disorder among patients seeking orthodontic treatment

**DOI:** 10.1186/s40510-020-00322-8

**Published:** 2020-08-03

**Authors:** Haritha Pottipalli Sathyanarayana, Sridevi Padmanabhan, R. Balakrishnan, Arun B. Chitharanjan

**Affiliations:** 1 Department of Orthodontics, Sri Ramachandra Dental College, Sri Ramachandra Institute of Higher Education and Research, Porur, Chennai, Tamil Nadu 600116 India; 2 Sri Ramachandra Medical Centre, Porur, Chennai, Tamil Nadu 600116 India; 3 Chennai, India

**Keywords:** Body Dysmorphic Disorder, Prevalence, Screening, Orthodontics

## Abstract

**Background:**

Body Dysmorphic Disorder (BDD) is a psychiatric disorder with delusions about defects in appearance for which patients seek various treatments. Patients with BDD often seek cosmetic procedures, and orthodontic treatment is one among them. This is the first Indian study to determine the prevalence of BDD in an orthodontic outpatient department.

**Materials and method:**

A total of 1184 patients with varying degrees of malocclusion completed the BDD-YBOCS questionnaire, while an experienced orthodontist assessed the severity of malocclusion with a rating scale.

**Results:**

Sixty-two patients (5.2%) were screened positive for BDD. Most of the BDD-positive patients were single (*p* value of 0.02) and had multiple previous consultations for orthodontic treatment (*p* value of < 0.00**) with a gender predilection toward males (*p* value of 0.00**), and age was not statistically significant with a *p* value of 0.3.

**Conclusion:**

From our study, the prevalence of BDD among orthodontic patients was 5.2%. The orthodontist should be aware of the high prevalence of BDD among orthodontic patients and identify the expectations of the patient at the time of history taking and refer the patient to a psychiatrist for diagnosis and appropriate management.

## Introduction

There has been an increase in the number of patients who seek orthodontic treatment to improve their appearance and quality of life because of the varied treatment options available. Body image plays an important role for patients requesting orthodontic treatment. Body Dysmorphic Disorder (BDD) previously known as dysmorphophobia is also referred to as body dysmorphia or dysmorphic syndrome. BDD was first described by Morselli as dysmorphophobia. In 1987, the American Psychiatric Association recognized this disorder as “Body Dysmorphic Disorder” in the Diagnostic and Statistical Manual of Mental Disorders IV (DSM IV) [1] and the International Classification of Diseases-10 [2] which is described as a mental condition characterized as a distressing or impairing preoccupation with an imagined or a slight defect in appearance.

According to DSM-5, BDD is now classified under Obsessive-Compulsive and related disorders requiring the presence of repetitive behaviors or mental acts in response to appearance concerns [3]. BDD was earlier considered as a somatoform disorder in which the affected person was excessively concerned about and preoccupied by a perceived defect in his or her physical features (body image). BDD is currently described as an Obsessive-Compulsive-related disorder since there is a pathological fear of ugliness in some parts of the body although no defect is observed by others or, if any, is thought to be slight [4].

The head and face seem to be the most concerned part of the body among BDD patients which includes acne, wrinkles, scars, vascular markings, swelling, facial asymmetry or disproportion, hair thinning, or excessive facial hair [5, 6]. Apart from the above mentioned concerns, BDD patients are also concerned about the nose, eyes, eyelids, eyebrows, ears, cheeks, teeth, lips, mouth, and jaws. Teeth whitening, jaw surgery, and braces were the major concerns for BDD patients to visit the general dentists. Orthodontic concerns were rotations, spacing, upper and lower midlines, and tooth size [7].

In patients who seek cosmetic therapy, BDD can be more prevalent and may remain unrecognized. Most of the BDD patients who undergo regular dental or orthodontic treatment are not satisfied with the results and seek other dentists and orthodontists for the same concern repeatedly. The psychological assessment and expectations of patients seeking orthodontic treatment is an important and therefore critical part of the overall assessment. It allows us to understand the potential problems at an initial stage before commencing the treatment.

BDD is reported in 2% of the general population and between 6 and 15% of dermatologic and cosmetic surgery patients in the USA [8]. Only two studies have been published on the prevalence of BDD among orthodontic patients. Hepburn and Cunningham conducted a study on 40 adult orthodontic patients and found that 7.5% of orthodontic patients were screened positive for BDD compared to 2.9% in a general public sample [9]. The second study by Yassaei et al. evaluated 270 Iranian orthodontic patients and reported that fifteen patients (5.5%) screened positive for BDD [10]. The prevalence of BDD in the dermatological setting was 8–15% [11, 12], and plastic surgery patients were 3–53% [13, 14]. As orthodontists are likely to encounter such patients in their practice, it is mandatory to understand and be familiar with BDD so that these patients could be referred to a psychiatrist for diagnosis and appropriate management.

Leone et al. [15] reported the diagnostic criteria as follows: (1) Preoccupation with an imagined defect in appearance. If a slight physical anomaly is present, the person’s concern is excessive. (2) The preoccupation causes clinically significant distress or impairment in social, occupational, or other important areas of functioning. (3) The preoccupation is not better accounted for by another mental disorder. BDD is also associated with significant occupational and social impairment and suicidal tendencies [16]. BDD is a poorly studied condition among Asian populations, and to our knowledge, no data exist about the prevalence of BDD among Indian orthodontic patients.

### Aim

The following are the aims of the present study:

1. To screen and identify the patients who are seeking orthodontic treatment for BDD
2. To establish an association between demographic variables and BDD in the study population

## Materials and method

This study was conducted in the Department of Orthodontics, Sri Ramachandra Institute of Higher Education and Research, Chennai, India. The study protocol was approved by the Institutional Ethics Committee (IEC-NI/12/MAR/27/15). The sample size with the power of 80 was calculated, and 1184 patients who had been referred to the Department of Orthodontics seeking orthodontic treatment participated in the study. The inclusion criteria were that the patient had to be over 18 years of age and willing to participate in the study and fill out the questionnaire. Patients with physical deformities, craniofacial syndromes, cleft lip and/or palate, and skeletal malocclusion requiring orthognathic surgery were excluded from the study. All the subjects were informed in detail about the questionnaire and had consented to participate in the study. All new patients who met the inclusion criteria received a self-report version of the BDD-YBOCS questionnaire which has reliable validity and reproducibility [17]. A modified self-report version of the BDD-YBOCS questionnaire has 12 questions of which the first three were related to the diagnostic criteria of BDD and the rest of the questions were focused on determining the severity of symptoms [18]. Diagnosis is based on the score of a patient in the first three questions. A score of 3 is suggestive of no BDD, while scores of 4–5 indicate mild BDD, 6–7 represent moderate BDD, and 8–9 and 10–11 pertain to severe and extremely severe BDD, respectively [19]. Age, gender, and marital status were added to the questionnaire. In addition, one question regarding the history of previous orthodontic consultations from the screening questionnaire for BDD in orthodontic patients suggested by Polo was included in our questionnaire [7]. Once the questionnaire was filled, an experienced orthodontist rated the severity of malocclusion in all the patients. The scale ranges from 1 to 4, where grade 1 is very extremely minor malocclusion; grade 2 is described as mild tooth rotations, mild inter-dental spacing, minimal misalignment of the midline, and other minor tooth imperfections; grade 3 is moderate or borderline need; and grade 4 definitely requires treatment.

The rating accuracy of the orthodontist was assessed using 25 photographs of patients with varying degrees of malocclusion, and the assessment was repeated after a 2-week interval which showed an intra-class correlation coefficient of 0.7.

### Statistical analyses

The data was analyzed using the Statistical Package for Social Sciences (SPSS) version 18 for Windows (SPSS, Inc., Chicago, IL, USA). The BDD-YBOCS questionnaire was used, and the patients were determined BDD-positive or BDD-negative. The mean age between BDD-positive and BDD-negative patients was compared using Student’s *t* test while the Chi-square test was used for the nominal variables such as the gender, marital status, and history of previous orthodontic consultations. The statistical significance was considered to be *p* ≤ 0.05.

## Results

From the 1184 patients, 62 patients (5.2%) of which 49 men and 13 women were diagnosed with BDD. Five were severely affected (BDD-YBOCS score 8–9), 19 were moderate (BDD-YBOCS score 6–7), and 38 were mild (BDD-YBOCS score 4–5) and is described in Table 1.

**Table 1:** Results of the BDD-YBOCS questionnaire

No. of patients (1184)	Percent	BDD-YBOCS score	Inference	Concerned
1122	94.8	≤ 3	No BDD	–
38	3.2	4–5	Mild BDD	Teeth
19	1.6	6–7	Moderate BDD	Teeth, hair
5	0.4	8–9	Severe BDD	Teeth, lips, nose, face

Comparisons between BDD-positive and BDD-negative patients which is described in Table 2 showed that BDD patients in our sample were younger with a mean age of 20.4 years than non-BDD patients with a mean age of 24.3 years but was not statistically significant (*p* = 0.3); among the 62 patients with BDD, 49 of them were males and 13 were females (*p* = 0.0003). Considering the marital status, 46 patients were single and 16 were married (*p* = 0.02). Most of the 44 BDD patients had sought previous orthodontic consultations before, and 18 patients reported for their first consultation (*p* = 0.000).

**Table 2:** Comparison of the questionnaire’s items between BDD and non-BDD patients

Variables		*N* = 1184	BDD-positive (62)	BDD-negative (1122)	
Age	Age	23.4 years	20.4 years	24.3 years	0.3^NS^
	Range	18–30 years	18–25 years	18–30 years	
Gender	Male	682 (58%)	49 (79%)	633 (56%)	0.0003***
	Female	502 (42%)	13 (21%)	489 (44%)	
Marital status	Single	699 (59%)	46 (74%)	653 (58%)	0.02*
	Married	485 (41%)	16 (32%)	469 (41%)	
Prior orthodontic consultation	No previous orthodontic consultation	1050 (89%)	18 (29%)	1032 (92%)	< 0.0**
	Previous orthodontic consultation	134 (11%)	44 (71%)	90 (8%)	

*NS* Not Significant

*p value < 0.05

**p ≤ 0.01

***p ≤ 0.001

Characteristics of the malocclusion in BDD-positive patients are described in Table 3. Of the 62 BDD-positive patients, 29 patients (47%) had minor tooth rotations, 17 patients (27%) had very mild spacing, nine patients (15%) had mild midline discrepancy, two patients (3%) had minimal tooth size imperfections, and five patients (8%) had other minimal tooth irregularities.

**Table 3:** Orthodontic concerns in BDD patients

Diagnoses	BDD patients (*n* = 62)	
Tooth rotations	29	47%
Very mild spacing	17	27%
Mild midline discrepancy	9	15%
Tooth size imperfections	2	3%
Other minimal irregularities	5	8%

## Discussion

This is the first Indian study to assess the prevalence of BDD among patients seeking orthodontic treatment. In our study, 62 patients (5.2%) among 1184 patients were screened positive for BDD. Among the patients who screened positive for BDD, the focus of concerns in 38 patients was on the teeth and 19 patients on the teeth and hair, and the preoccupations in the other five patients included teeth, lips, nose, and pigmentation on the face. In our study, BDD-positive patients were concerned about very mild rotations, spacing, midline discrepancy, and tooth size discrepancies which did not require orthodontic treatment. The age group of our patients included in the study was over 18 years since symptoms of BDD usually begin during adolescence [20]. In our study, BDD patients were younger than non-BDD patients, but it was not statistically significant. This finding was in contrary to the findings of Uzun et al. [21] but similar to the findings of Yassaei et al. [10], Vulink et al. [22], and Philips et al. [23] in which BDD was more prevalent in the younger age group.

A gender preference toward males was seen in our study which also accords with some earlier findings [22] and is in contrast with others [10, 23]. Philips et al. concluded that there was a gender ratio of 1.27 for women to men in the community and 1.64 among students whereas there was an equal gender distribution in psychiatric service. According to the American Society of Plastic Surgeons in 2014, the gender ratio is reversed in general cosmetic surgery and in rhinoplasty settings with more prevalence among men. Considering the above statistics, our study showed more prevalence among men [24, 25].

In our study, the majority of the BDD patients were single which correlates with the findings of Hepburn and Cunningham [9]. One question suggested by Polo was added which was not the repetition of BDD-YBOCS questions and had the following answer: yes or no [7]. Our BDD patients had multiple previous orthodontic consultations which were in accordance with the study of Yassaei et al. [10]. Hepburn and Cunningham conducted an interview-based study and concluded that BDD was diagnosed in 2 patients (2.86%) among 70 members of the general public and 3 patients (7.5%) among 40 adult orthodontic patients [9]. Vulink et al. concluded that the prevalence of BDD in a maxillofacial outpatient setup presenting for orthognathic surgery for a period of 6 months was 17% [23]. The prevalence of BDD among Indian orthodontic patients seems to be less than other populations. Differences in the ethnicity of study population, inclusion and exclusion criteria, self-report questionnaire, or an interview with a psychiatrist could play an important role in varied results. A questionnaire is one of the most convenient and proven ways to screen and identify patients who may suffer from BDD. In order to correctly diagnose BDD, a series of questions are asked to the patient to determine if they are consumed with distress about a seemingly small or unnoticeable flaw. In accordance with previous studies, the head and face seem to be the common areas of concern among BDD patients [5, 6]. However, all patients were assessed by a senior orthodontist as having mild malocclusions, which was more likely to be accepted by most people, or no obvious malocclusion was present.

BDD affects the quality of life and is associated with depression and Obsessive-Compulsive disorder. It is important to understand the expectations and also to know whether a patient has previously received the treatment. It is essential to elicit the age at which the concerns started and its impact on their lives. During history taking and clinical examination, additional time should be spent on each patient assessing their need for treatment, and questions to identify patients with BDD should also be included. The questionnaire can serve as a tool in screening patients who might be a suspect of BDD. Orthodontists should refer the patients who are suspicious with a slight defect insisting on treatment or an imagined defect to a psychiatrist before initiating orthodontic treatment. A psychiatrist plays an important role in helping us to identify these patients as well as treating them with pharmacotherapy and behavioral therapy.

## Conclusion

The following conclusions were drawn from our study:

1. This study suggests that BDD occurs in adult orthodontic patients, and its prevalence was 5.2% in our sample of 1184 patients. The teeth and face seem to be the most common areas of concern.
2. BDD-positive patients in our study population were mostly males, single, younger, and had previous orthodontic consultations than BDD-negative patients.
3. Orthodontists should be aware of its features and include a few questions during history taking to help identify potentially high-risk patients.

## Supplementary information

**Additional file 1.** Questionnaire.

## Data Availability

From the corresponding author

## References

[1] American Psychiatric Association (2000). Diagnostic and statistical manual of mental disorders. Fourth Edition Text Revision.

[2] World Health Organization (1992). The ICD-10 Classification of Mental and Behavioural Disorders.

[3] American Psychiatric Association (2013). Diagnostic and statistical manual of mental disorders.

[4] Ahluwalia R, Bhatia NK, Kumar PS, Kaur P (2017). Body dysmorphic disorder: diagnosis, clinical aspects and treatment strategies. Indian J Dent Res.

[5] American Psychiatric Association (1994). Diagnostic and statistical management of mental disorders.

[6] Phillips KA, Dufresne RG (2000). Body dysmorphic disorder. A guide for dermatologists and cosmetic surgeons. Am J Clin Dermatol.

[7] Polo M (2011). Body dysmorphic disorder: a screening guide for orthodontists. Am J Orthod Dentofacial Orthop.

[8] Wilson JB, Arpey CJ (2004). Body dysmorphic disorder: suggestions for detection and treatment in a surgical dermatology practice. Dermatol Surg.

[9] Hepburn S, Cunningham S (2006). Body dysmorphic disorder in adult orthodontic patients. Am J Orthod Dentofacial Orthop.

[10] Yassaei S, Goldani Moghadam M, Aghili H, Tabatabaei SM (2014). Body dysmorphic disorder in Iranian orthodontic patients. Acta Med Iran.

[11] Dufresne RG, Phillips KA, Vittorio CC, Wilkel CS (2001). A screening questionnaire for body dysmorphic disorder in a cosmetic dermatologic surgery practice. Dermatol Surg.

[12] Phillips KA, Dufresne RG, Wilkel CS, Vittorio CC (2000). Rate of body dysmorphic disorder in dermatology patients. J Am Acad Dermatol.

[13] Aouizerate B, Pujol H, Grabot D, Faytout M, Suire K, Braud C (2003). Body dysmorphic disorder in a sample of cosmetic surgery applicants. Eur Psychiatry.

[14] Vindigni V (2002). The importance of recognizing body dysmorphic disorder in cosmetic surgery patients: do our patients need a preoperative psychiatric evaluation?. Eur J Plast Surg.

[15] Leone JE, Sedory EJ, Gray KA (2005). Recognition and treatment of muscle dysmorphia and related body image disorders. J Athl Train.

[16] Phillips KA (1999). Body dysmorphic disorder and depression: theoretical considerations and treatment strategies. Psychiatr Q.

[17] Phillips KA, Hollander E, Rasmussen SA (1997). A severity rating scale for body dysmorphic disorder: development, reliability, and validity of a modified version of the Yale-Brown obsessive compulsive scale. Psychopharmacol Bull.

[18] Phillips KA (2009). Understanding body dysmorphic disorder; an essential guide.

[19] Philips KA (1996). The broken mirror: understanding and treating BDD.

[20] Phillips KA (1998). Body dysmorphic disorder: clinical aspects and treatment strategies. Bull Menninger Clin.

[21] Uzun O, Basoglu C, Akar A (2003). Body dysmorphic disorder in patients with acne. Compr Psychiatry.

[22] Vulink NCC, Rosenberg A, Plooij JM, Koole R, Bergé SJ, Denys D (2008). Body dysmorphic disorder screening in maxillofacial outpatients presenting for orthognathic surgery. Int. J. Oral Maxillofac. Surg..

[23] Phillips KA, Menard W, Fay C (2005). Demographic characteristics phenomenology, comorbidity, and family history in 200 individuals with body dysmorphic disorder. Psychosomatics.

[24] Phillips KA, Didie ER, Menard W, Pagano ME, Fay C, Weisberg RB (2006). Clinical features of body dysmorphic disorder in adolescents and adults. Psychiatry Research.

[25] American Society of Plastic Surgeons (2014). Plastic surgery statistics report. ASPS National Clearinghouse of Plastic Surgery Procedural Statistics.

