# Supplementary material for: Prevalence of Body Dysmorphic Disorder among patients seeking orthodontic treatment
Source: Prog Orthod. 2020 Aug 3;21:20. doi: 10.1186/s40510-020-00322-8 (PMC7396409; doi:10.1186/s40510-020-00322-8)
Supplement: Supplementary file 1 — Additional file 1. Questionnaire. [file 40510_2020_322_MOESM1_ESM.docx]

Additional file 1: **QUESTIONNAIRE**

1. **Are you very concerned about the appearance of some part of your body which you consider especially unattractive?**

Yes/No

(If yes, continue with the next question)

1. **Does the face represent the main area of your body you consider as unattractive**

Yes/No (If yes, continue with the next question)

1. **Which part of the face you consider as unattractive? (Kindly tick in order of priority)**
2. Shape
3. Eyes
4. Nose
5. Lips
6. Smile
7. Teeth
8. Jaws
9. Skin
10. **Kindly give details of your dissatisfaction with the first two in your priority list.**
11. **Is this your first visit to a dentist or orthodontist regarding this problem?**

BDD- Body Dysmorphic Disorder
